# Stat3 Activates the Receptor Tyrosine Kinase Like Orphan Receptor-1 Gene in Chronic Lymphocytic Leukemia Cells

**DOI:** 10.1371/journal.pone.0011859

**Published:** 2010-07-29

**Authors:** Ping Li, David Harris, Zhiming Liu, Jie Liu, Michael Keating, Zeev Estrov

**Affiliations:** Department of Leukemia, The University of Texas M. D. Anderson Cancer Center, Houston, Texas, United States of America; Roswell Park Cancer Institute, United States of America

## Abstract

**Background:**

The receptor tyrosine kinase like orphan receptor (ROR)-1 gene is overexpressed in chronic lymphocytic leukemia (CLL). Because Stat3 is constitutively activated in CLL and sequence analysis revealed that the ROR1 promoter harbors γ-interferon activation sequence-like elements typically activated by Stat3, we hypothesized that Stat3 activates ROR1.

**Methodology/Principal Findings:**

Because IL-6 induced Stat3 phosphorylation and upregulated Ror1 protein levels in MM1 cells, we used these cells as a model. We transfected MM1 cells with truncated ROR1 promoter luciferase reporter constructs and found that IL-6 induced luciferase activity of ROR1-195 and upstream constructs. Co-transfection with Stat3 siRNA reduced the IL-6-induced luciferase activity, suggesting that IL-6 induced luciferase activity by activating Stat3. EMSA and the ChIP assay confirmed that Stat3 binds ROR1, and EMSA studies identified two Stat3 binding sites. In CLL cells, EMSA and ChIP studies determined that phosphorylated Stat3 bound to the ROR1 promoter at those two ROR1 promoter sites, and ChIP analysis showed that Stat3 co-immunoprecipitated DNA of STAT3, ROR1, and several Stat3-regulated genes. Finally, like STAT3-siRNA in MM1 cells, STAT3-shRNA downregulated STAT3, ROR1, and STAT3-regulated genes and Stat3 and Ror1 protein levels in CLL cells.

**Conclusion/Significance:**

Our data suggest that constitutively activated Stat3 binds to the ROR1 promoter and activates ROR1 in CLL cells.

## Introduction

Receptor tyrosine kinase like orphan (Ror) proteins are type-I transmembrane receptor tyrosine kinase members of the neurotrophic tyrosine kinase receptor superfamily that regulate the developmental wingless-type (Wnt) signaling pathway [1]. The extracellular region of Ror proteins contains an immunoglobulin domain, a Cys-rich domain, also called the Frizzled domain, and a Kringle domain. Intracellularly, Ror proteins possess a tyrosine kinase domain and a proline-rich domain straddled by two Ser/Thr-rich domains [2]. The vertebrate ROR family members, ROR1 and ROR2, were first identified in a human neuroblastoma cell line by a polymerase chain reaction (PCR)-based search for tyrosine kinases [2]. In humans, Ror protein functions are involved in skeletal development. Human ROR2 mutations cause well-characterized skeletal defects such as dominant brachydactily type B, a condition of shortened or missing digits [3], [4], and recessive Robinow syndrome, a form of short-limbed dwarfism [5], [6]. Human ROR2 polymorphisms are also associated with variations in bone length and mineral density [7]. Similar to ROR2, human ROR1 plays a role in embryonic organogenesis. However, unlike ROR2, mutations in human ROR1 have not been linked to any human disease thus far.

ROR1, however, is overexpressed in B-cell chronic lymphocytic leukemia (CLL) cells. Unlike normal granulocytes, B-, or T-cells, leukemia cells from all patients with CLL spontaneously express ROR1 mRNA [8], [9], [10]. Protein analysis has revealed that CLL blood lymphocytes, but not normal resting B-lymphocytes, express two different variants of the Ror1 protein, 105 kd and 130 kd [8]. Cell-surface Ror1 has been detected in CLL cells by flow cytometry, western immunoblotting, and ELISA [10]. Anti-Ror1 monoclonal antibodies were found to react with CLL cells, but not with a variety of normal adult tissues, nonleukemic leukocytes, or blood mononuclear cells, including CD5+ B cells of healthy adults [11].

**Figure 1:**
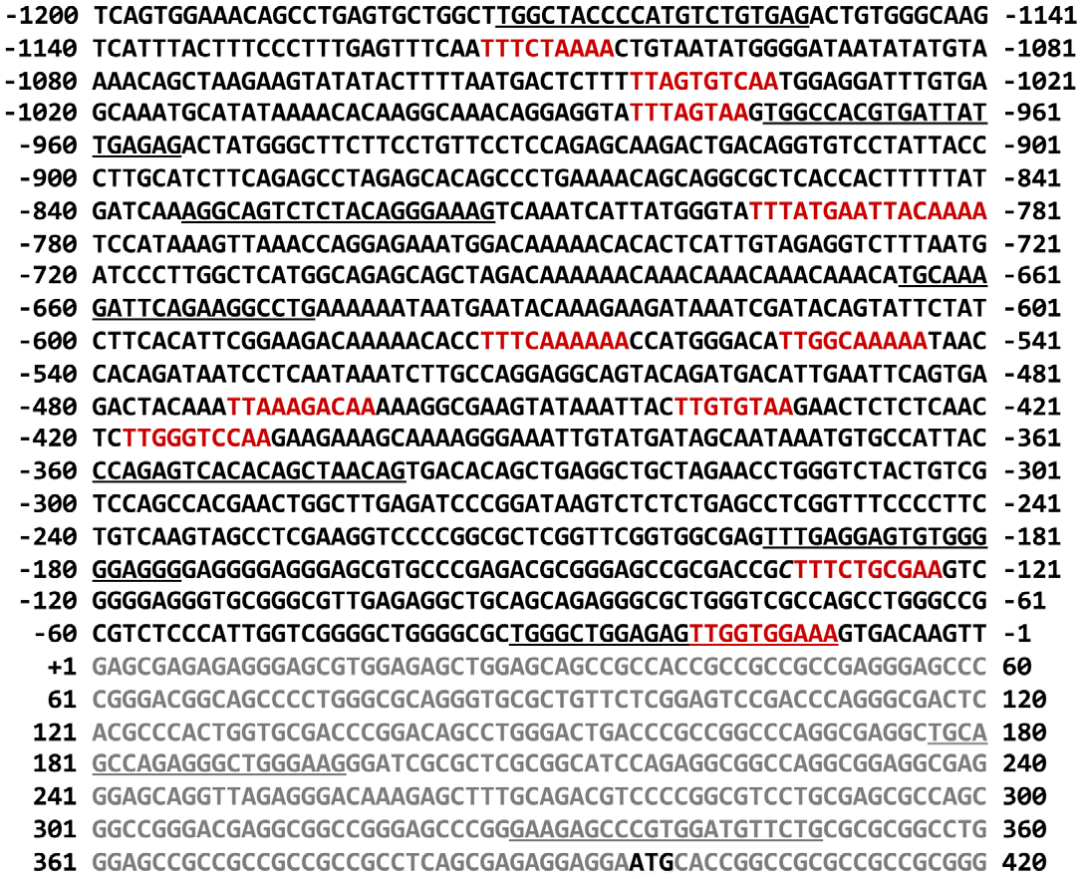
Sequence of the 5′-flanking region of the human ROR1 promoter. Underlined are the primers used to generate ROR1 promoter fragments. The putative Stat3 binding sites are shown in red. Gray denotes the exon region and ATG is the starting codon.

B-cell CLL, the most common adult leukemia in the Western hemisphere, is characterized by the accumulation of neoplastic B-lymphocytes co-expressing CD5 and CD19 antigens [12]. Unlike in other hematologic malignancies, in CLL Stat3 is constitutively phosphorylated exclusively on serine 727 residues [13], translocates to the nucleus, binds to DNA, and activates the transcription of genes known to be transcribed by tyrosine-phosphorylated Stat3 [14]. Typically, phosphorylated Stat3 binds to the γ-interferon activation sequence (GAS)-like element, also referred to as the sis-inducible element, located in the promoter region of various genes [15]. Because sequence analysis identified GAS-like elements in the ROR1 promoter (Fig. 1), we hypothesized that Stat3 binds to the ROR1 promoter in CLL cells.

## Results

### CLL cells express Ror1 protein

**Figure 2:**
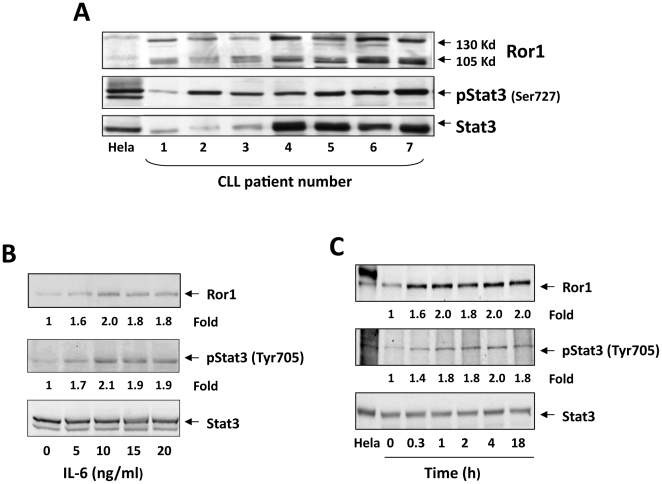
CLL cells constitutively express phosphoserine-Stat3 and Ror1 protein, and IL-6 upregulates phosphotyrosine-Stat3 and Ror1 protein levels in MM1 cells. **A**. Western immunoblotting of unstimulated CLL cells from 7 different patients shows constitutive expression of phosphoserine-Stat3 and both the 105- and 130-Kd variants of the Ror1 protein. **B**. MM1 cells were incubated for 2 hours with increasing concentrations of IL-6. As determined by western immunoblotting, IL-6 induced Stat3 tyrosine phosphorylation and upregulated Ror1 protein levels in a dose-dependent manner. **C**. MM1 cells were incubated with 20 ng/ml IL-6 and harvested at different time points for further analysis. As determined by western immunoblotting, IL-6 induced Stat3 tyrosine phosphorylation and upregulated Ror1 protein levels in a time-dependent manner.

We first sought to confirm that Ror1 protein is present in unstimulated CLL cells [8], [10], [11]. We fractionated low-density cells from the peripheral blood (PB) of seven patients with CLL (Table S1), and by using western immunoblotting, we detected both the 105- and 130-Kd variants of the Ror1 protein in all samples. As expected [13], [14], we also found that Stat3 was constitutively phosphorylated on serine 727 residues in all samples (Fig. 2A).

### IL-6 phosphorylates Stat3 and upregulates Ror1 protein levels in MM1 cells

To test the hypothesis that Stat3 activates ROR1, we used the multiple myeloma cell line MM1. Unstimulated MM1 cells express low to undetectable levels of phosphotyrosine-Stat3, and exposure of MM1 cells to IL-6 induces Stat3 phosphorylation [16], [17]. We incubated MM1 cells with IL-6 and, using western blot analysis, found that IL-6 induced tyrosine phosphorylation of Stat3. IL-6 also upregulated Ror1 protein levels in a dose-and time-dependent manner, whereas both phospho-Stat3 and Ror1 were almost undetectable in untreated MM1 cells (Fig. 2B).

### IL-6 induces ROR1 promoter activity in MM1 cells by activating Stat3

**Figure 3:**
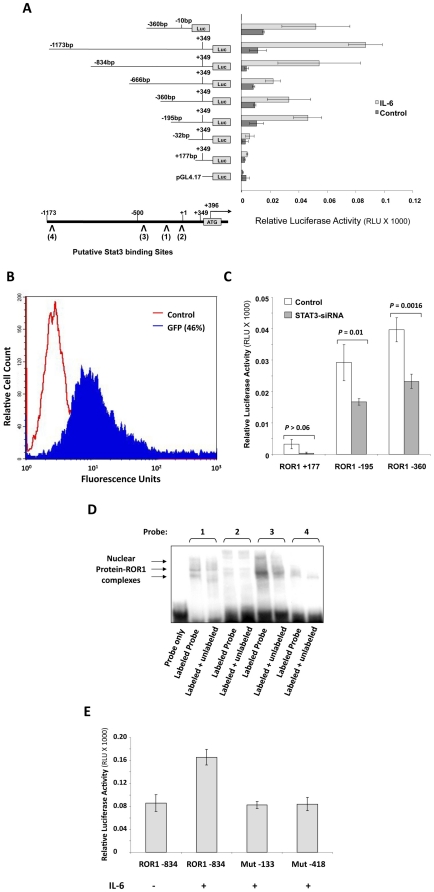
IL-6 induces ROR1 promoter activity by activating Stat3. **A**. A schematic diagram of the ROR1 reporter constructs (left) that were transfected into MM1 cells and maintained in the absence or presence of 20 ng/ml IL-6; their relative luciferase activity is also depicted. The right panel depicts the means ± standard deviation of the relative luciferase activity measured in four different experiments in which transfection efficiency of the truncated ROR1 promoter constructs into MM1 cells was at least 40%. Construct ROR1 −195 and the upstream constructs exhibited significant IL-6-induced luciferase activity. The schematic diagram in the left lower corner depicts four putative Stat3 binding sites. **B**. Co-transfection efficiency of IL-6-stimulated MM1 cells previously transfected with the ROR +177, ROR1 −195, and ROR1 −360 truncated constructs. **C**. STAT3-siRNA downregulated the luciferase activity of MM1 cells transfected with ROR1 −195 and ROR1 −360 but not ROR1 +177, suggesting that IL-6 induced luciferase activity by activating Stat3. Student's *t* test was used to determine whether the differences between luciferase activity values are significant. **D**. EMSA using biotin-labeled and unlabeled probes of the four putative Stat3 binding sites shows that MM1 cell nuclear protein bound to the biotinylated DNA probes 1 and 3 and that the addition of the corresponding cold (unlabeled) probes attenuated the binding of the hot (labeled) probes. Binding to probe 1 is lighter than probe 3 because of unequal loading. **E**. Single nucleotide (T to G) mutation either at position −133 or position −418 abrogates the IL-6-induced luciferase activity of ROR1 −834.

In order to determine whether activated Stat3 binds to the ROR1 promoter, we cloned the human ROR1 promoter (1522 bp) into a luciferase reporter plasmid and generated a series of truncated constructs (Fig. 3A, left panel). The location of the 5′ regions of these constructs is underlined in Fig. 1, and the primers used to generate them are depicted in Table S2. All constructs were transfected into MM1 cells (transfection efficiency: 45–50%; not shown), and their activity was assessed, after incubation with either 20 ng/ml IL-6 or culture medium, by using the luciferase reporter assay and the Renila system. Exposure of MM1 cells to IL-6 significantly increased luciferase activity in ROR1 −195 (from −195 to +349; 544 bp), ROR1 −666 (from −666 to +349; 1015 bp), ROR1 −834 (from −834 to +349; 1183 bp), and ROR1 −1173 (from −1173 to +349; 1522 bp). IL-6 did not significantly increase luciferase activity in ROR1 −32 (from −32 to +349; 381 bp) or ROR1 +177 (from +177 to +349; 172 bp) (Figure 3A, right panel). Among the serial deletion clones, ROR1 −1173 exerted the highest, and ROR1 −32 the lowest, IL-6-induced promoter activity (Fig. 3A).

The luciferase activity of ROR1 −195, ROR1 −360 (from −360 to +349; 709 bp), ROR1 −666, and ROR1 −834 suggests that the ROR1-activating elements are located upstream of −32 bp and that the region spanning between −666 and −360 bp contains one or more negative regulatory elements. Because the region downstream of −195 contains two GAS-like elements, and ROR1 −32 had no luciferase activity, we assumed that STAT3 binds to a GAS-like element located at minus 133 to −124 bp (Fig. 1). To test this assumption, a construct of 350 bp spanning from −360 to −10 was prepared. The luciferase activity of this construct was almost identical to that of ROR1 −1173 (Fig. 3A), confirming that this region plays a crucial role in ROR1 expression and suggesting that −133 to −124 bp is a Stat3 binding site.

These results, together with the data showing that IL-6 induced Stat3 phosphorylation and upregulated Ror1 protein levels in MM1 cells (Fig. 2B), suggest that IL-6 induces luciferase activity of the ROR1 promoter by activating Stat3. To further delineate these findings, MM1 cells were co-transfected with the ROR1 −195 or ROR1 −360 construct and Stat3-siRNA (co-transfection efficiency: 46%; Fig. 3B) and incubated with IL-6. As shown in Fig. 3C, co-transfection with Stat3-siRNA significantly downregulated the IL-6-induced luciferase activity of ROR1 −195 or ROR1 −360, confirming that IL-6 induced luciferase activity by activating Stat3.

### Stat3 binds to the ROR1 promoter and activates ROR1 in MM1 cells

Because we assumed that the Stat3-binding sites are located at −133 to −124 bp and upstream regions such as −429 to −400 bp (Fig. 3A, lower panel; Fig. 1), we generated four biotinylated DNA probes, each harboring a putative Stat3 binding site (one downstream [probe 2], and two upstream [probes 3 and 4] of the region spanning from bp −133 to −124 [probe 1]) and, using these probes, we conducted an EMSA. As shown in Fig. 3D, probes 1 and 3, but not probes 2 and 4, bound IL-6-stimulated MM1-cell nuclear extract, and the binding of the labeled probes was reduced by the corresponding unbiotinylated probe (Fig. 3D), suggesting that nuclear protein binds to these sites. Indeed, a T to G mutation either at position −133 or position −418 of these putative Stat3 binding sites abrogated the IL-6-induced luciferase activity of ROR1 −834 (Fig. 3E).

**Figure 4:**
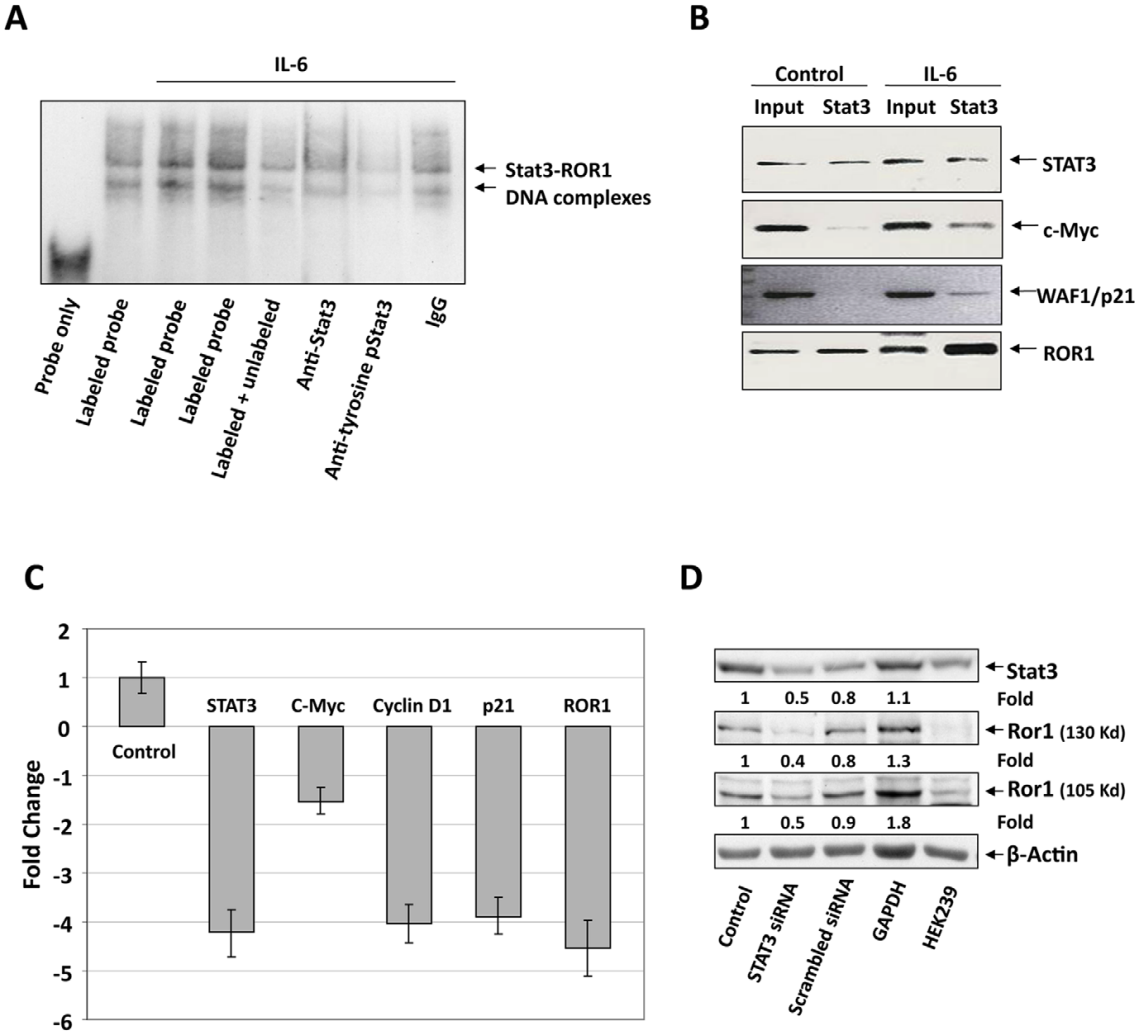
IL-6-activated Stat3 binds to the ROR1 promoter and activates transcription in MM1 cells. **A**. ROR1 promoter biotinylated DNA probe binds to nuclear protein of unstimulated or IL-6-stimulated MM1 cells. The addition of an unlabeled (cold) probe attenuated the binding of the hot (biotin-labeled) probe. Similarly, anti-Stat3 (total Stat3) and anti-phosphotyrosine-Stat3 antibodies attenuated the binding of the hot probe, suggesting that nuclear Stat3 protein bound the labeled ROR1 probe. **B**. ChIP demonstrates that anti-(total) Stat3 antibodies immunoprecipitated ROR1, STAT3, and the Stat3-regulated genes c-Myc and p21. These genes were detected in MM1 cell nuclear extracts (Input) as well as in nuclear protein immunoprecipitated with anti-Stat3 antibodies, particularly in IL-6-stimulated MM1 cells, suggesting that Stat3, known to bind Stat3-regulated gene promoters, also binds the ROR1 promoter. **C**. qRT-PCR demonstrates that STAT3-siRNA downregulates the mRNA levels of ROR1, STAT3, and the Stat3-regulated genes c-Myc, Cyclin D1, and p21. S18 mRNA (control) was not affected. **D**. Western blot analysis shows that STAT3-siRNA, but not scrambled-siRNA or GAPDH, downregulates the protein levels of Stat3 and Ror1.

Then, to determine whether Stat3 is the nuclear protein that binds to the ROR1 promoter, we conducted another EMSA using probe 1. As shown in Fig. 4A, the biotinylated ROR1 DNA probe bound MM1 nuclear protein, and anti-Stat3 and anti-phosphotyrosine Stat3 antibodies attenuated the nuclear protein-DNA binding, suggesting that activated Stat3 binds to the ROR1 promoter. To validate these findings, we conducted a ChIP analysis of IL-6-treated and -untreated MM1 cells. As shown in Fig. 4B, anti-Stat3 antibodies immunoprecipitated ROR1, STAT3 (as Stat3 binds to and induces STAT3 [18]), the Stat3-regulated gene WAF1/p21, and c-Myc promoters. A stronger signal was obtained from chromatin of IL-6-stimulated MM1 cells. Next, we sought to determine whether Stat3 transcribes ROR1. We transfected MM1 cells with STAT3-siRNA, stimulated them with IL-6, and determined mRNA levels of ROR1, STAT3, and various Stat3-regulated genes by using relative qRT-PCR. As shown in Fig. 4C, STAT3-siRNA downregulated mRNA levels of ROR1 and the Stat3-regulated genes STAT3, c-Myc, Cyclin D1, and WAF1/p21. Furthermore, STAT3-siRNA, but not scrambled siRNA or glyceraldehyde-3-phosphate dehydrogenase (GAPDH), downregulated the Stat3 protein levels by 50% and the 130-Kd and 105-Kd Ror1 protein variant levels by 60% and 50%, respectively, suggesting that Stat3 transcribes ROR1 and induces the production of Ror1 protein in MM1 cells (Fig. 4D).

### Serine-phosphorylated Stat3 binds the ROR1 promoter in CLL cells

**Figure 5:**
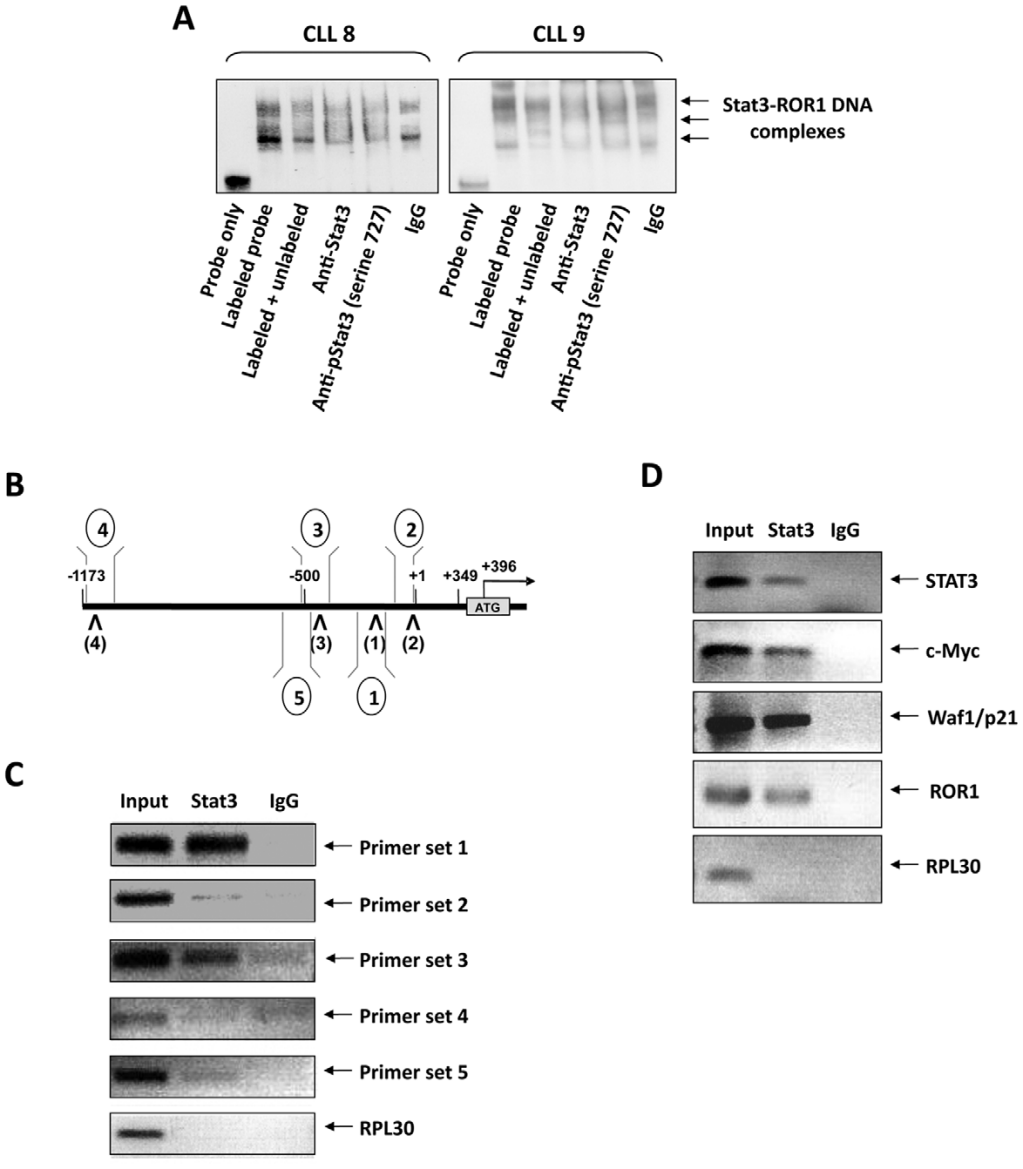
CLL-cell constitutively activated Stat3 binds the ROR1 promoter at the previously identified Stat3 binding sites. **A**. EMSA, performed using cells of CLL patients 8 and 9, demonstrates that CLL-cell nuclear protein binds the ROR1 biotinylated (labeled)-DNA probe. The cold (unlabeled) probe attenuated the binding of the hot probe and a similar effect was obtained with anti-Stat3 and anti-phosphoserine-Stat3 antibodies. IgG was used as control. These data suggest that Stat3 binds the ROR1 promoter in primary CLL cells. **B**. A schematic diagram showing the DNA regions for which 5′ and 3′ primers were designed to generate DNA probes, as described in the Materials and Methods. Primer sets 1 through 4 were designed to cover Stat3 putative binding sites (as shown in Fig. 3A), whereas primer set 5 covers a region lacking GAS-like elements (control). **C**. DNA probes generated by primer sets 1 and 3 (generating DNA probes harboring the corresponding Stat3 binding sites), but not those generated by primer sets 2, 4, or 5, bind nuclear protein of cells obtained from CLL patient 10, as assessed using ChIP. **D**. ChIP of nuclear protein from CLL cells of patient 11 demonstrates that Stat3 co-immunoprecipitated with ROR1, STAT3, and the Stat3-regulated genes c-Myc and p21. The control gene RPL-30, detected in whole-cell nuclear protein (Input), does not co-immunoprecipitate with Stat3.

In CLL cells Stat3 is constitutively phosphorylated on serine 727 residues and activates the transcription of Stat3-regulated genes [14]. To test whether Stat3 binds to the ROR1 promoter in CLL cells, we used EMSA. As shown in Fig. 5A, nuclear protein from two different patients bound to the ROR1 DNA biotinylated probe, and the binding was partially reversed by the unbiotinylated probe. Similarly, anti-Stat3 and anti-phosphoserine-Stat3 antibodies attenuated the protein-DNA binding (Fig. 5A). To determine whether Stat3 binds to the same binding sites in CLL cells as in MM1 cells, we used the ChIP assay. We generated five specific primer sets for PCR analysis (Fig. 5B). Four sets were designed to amplify promoter regions containing Stat3 putative binding sites that we previously studied in MM1 cells (Fig. 3), and an additional set (primer set 5) was designed to amplify a region lacking GAS-like elements. As shown in Fig. 5C, amplification was detected only with probes designed to amplify regions harboring the Stat3 binding sites 1 and 3, suggesting that these two Stat3 binding sites are operative in both MM1 and CLL cells. To confirm this observation, we conducted another ChIP study and found that Stat3 co-immunoprecipitated the promoters of ROR1 and the Stat3-regulated genes STAT3, c-Myc, and WAF1/P21 but not of the control gene RPL30 (Fig. 5D). These data suggest that Stat3 binds to the ROR1 promoter in CLL cells.

### Stat3 transcribes ROR1 and induces Ror1 protein production in CLL cells

**Figure 6:**
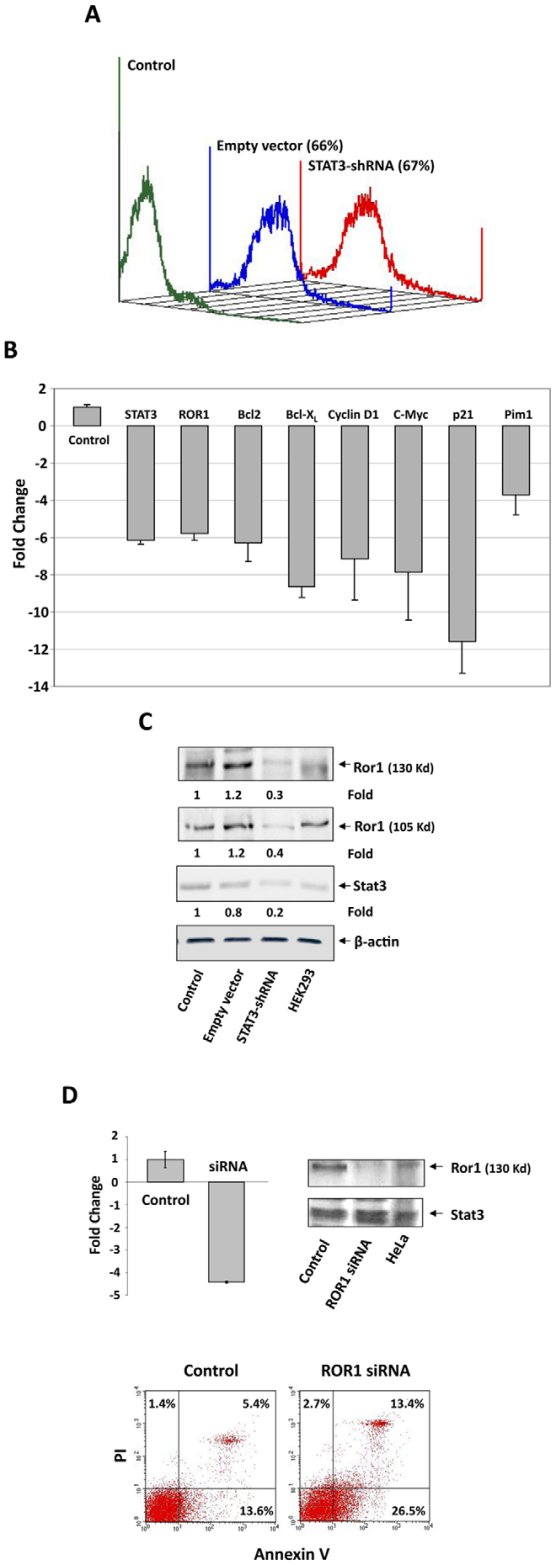
STAT3-shRNA downregulates ROR1 expression and Ror1 protein levels and ROR1-siRNA downregulates ROR1 and induces the apoptosis. **A**. A 67% infection efficiency of retroviral STAT3-shRNA into CLL cells from a patient was obtained. **B**. qRT-PCR of these CLL cells demonstrates that STAT3-shRNA downregulated mRNA levels of ROR1, STAT3, and the Stat3-regulated genes Bcl2, Bcl-X_L_, Cyclin D1, c-Myc, p21, and Pim1. S18 mRNA (control) was not affected. **C**. STAT3-shRNA, but not the empty vector, downregulates Stat3 and Ror1 protein levels. **D**. Left upper panel: ROR1-siRNA downregulated ROR1 mRNA levels. qRT-PCR results are depicted. GAPDH-siRNA was used as control. Right upper corner: ROR1-siRNA, but not GAPDH-siRNA, downregulates Ror1 protein levels. Lower panel: Knock-down of ROR1 induces apoptosis. ROR1-siRNA increased CLL cell apoptosis rate of from 19% (GAPDH-siRNA control) to 39.9% (ROR1-siRNA).

We next sought to determine whether Stat3 activates ROR1 in CLL cells. We infected CLL cells with a lentivirus harboring STAT3-shRNA (infection efficiency: 68%; Fig. 6A) and quantitated Stat3-regulated gene mRNA levels by relative qRT-PCR. As shown in Fig. 6B, infection with STAT3-shRNA reduced the mRNA levels of ROR1 and the Stat3-regulated genes STAT3, Bcl2, Bcl-X_L_, Cyclin D1, c-Myc, WAF1/p21, and Pim1. Furthermore, unlike empty vector, STAT3-shRNA downregulated the protein levels of Stat3 by 80% and of the 130-Kd and 105-Kd Ror1 protein variants by 60% and 70%, respectively (Fig. 6C). Remarkably, transfection of CLL cells with ROR1-siRNA downregulated ROR1 mRNA and Ror1 protein levels and induced apoptosis (Fig. 6D). Taken together, these data suggest that in CLL cells, Stat3, constitutively phosphorylated on serine 727 residues, activates ROR1 transcription and induces Ror1 protein production.

## Discussion

CLL is characterized by a dynamic imbalance between the proliferation and apoptosis of neoplastic B-lymphocytes co-expressing CD5 and CD19 antigens [19], [20], [21]. More than a decade ago, Frank et al. reported that Stat3 is constitutively phosophorylated on serine 727 residues in CLL cells [13]. Recently, we found that similar to tyrosine-phosphorylated Stat3 in other neoplasms, serine-phosphorylated Stat3 activates proliferation and survival genes in CLL [14]. Other investigators have reported that the Wnt signaling pathway is activated [22] and the Wnt receptor ROR1 gene is overexpressed in CLL cells. Here, we demonstrate that constitutively activated Stat3 binds to the ROR1 promoter, activates ROR1, and induces the production of Ror1 protein in CLL cells.

Wnt proteins are a large family of secreted glycoproteins that activate signal transduction pathways that control proliferation, survival, and migration. Gene expression analysis of CLL cells showed that multiple Wnt genes are highly expressed in CLL cells compared with normal B cells [9], [22]. Different factors contribute to Wnt gene expression in CLL. Wnt10b, transcribed by nuclear factor (NF)-κB [23], and Wnt5a, transcribed by NF-κB and the Notch signaling cascade [24], are both constitutively activated in CLL [25], [26]. In addition, several Wnt antagonist genes, such as WIF1, DKK3, APC, SFRP1, SFRP2, SFRP4, and SFRP5, are methylated and their expression downregulated in CLL cells [27], [28]. Because Wnt3 has been shown to promote the proliferation of mouse bone marrow pro-B cells [29], it was speculated that Wnt might stimulate CLL cell proliferation in an autocrine manner [9]. Indeed, an analog of a non-steroidal anti-inflammatory drug (R-etodolac) that inhibits the Wnt/β-catenin pathway induced apoptosis of CLL cells [22], and inhibition of Wnt by amides of ethacrynic acid decreased CLL cell survival [30]. Wnts are capable of signaling through several pathways, including the Wnt receptor Frizzled, which is reported to be overexpressed in CLL [9], [22], and the canonical β-catenin-mediated pathway [31], [32]. Remarkably, alternative splicing causing silencing of E-cadherin, a negative regulator that binds β-catenin and prevents its translocation to the nucleus [33], was recently described in CLL cells [34].

Ror proteins have emerged as central regulators of Wnt signaling [1]. ROR1 is overexpressed in CLL and, as confirmed in our current study, its protein is present in unstimulated CLL cells [8], [9], [10], [11] and its downregulation enhances apoptosis. Stat3 is constitutively activated in CLL [14], and activated Stat3 binds target gene promoters containing a GAS-like element [15]. Stat dimers recognize 8- to 10-base-pair inverted repeat DNA elements with a consensus sequence of 5′-TT(N_4–6_)AA-3′. Binding affinity of an activated Stat dimmer to a target DNA sequence is determined by variations in the nucleotide sequence [35] and by cooperative dimer-dimer interactions mediated by NH_2_-terminal amino acids [36], [37]. Because sequence analysis revealed that the ROR1 promoter harbors GAS-like elements, we asked whether Stat3 binds to the ROR1 promoter and induces Ror1 protein production in CLL cells.

That Stat3 activates ROR1 has not been previously reported. To study this interaction, we used, as a Stat3-inducible model, MM1 cells. Consistent with our previous study [16], we found that IL-6 induced Stat3 phosphorylation in MM1 cells and, as we assumed, upregulated Ror1 protein levels in a time- and dose-dependent manner. In order to identify Stat3 binding sites in the ROR1 promoter, we obtained genomic DNA from patients with CLL and cloned the ROR1 promoter. We generated different-length ROR1 promoter constructs, transfected them into MM1 cells, and by assessing their luciferase activity, found that IL-6 induced activity with constructs upstream of ROR1 −32. By using siRNA, we confirmed that Stat3 activates ROR1 promoter activity, and using EMSA we identified two Stat3 binding sites in the ROR1 promoter. One site is located within the region from −143 to −114 and the other from −429 to −400. Remarkably, MM1 cells transfected with ROR1 −666, which harbors the −429 Stat3 binding site, exhibited relatively low luciferase activity upon IL-6 stimulation, suggesting that a repressor(s) might be present in this region. Transfection of MM1 cells with STAT3-siRNA downregulated ROR1 mRNA and the protein levels of both Stat3 and Ror1, confirming that Stat3 induces the expression of ROR1 in MM1 cells.

ROR1 is overexpressed in various neoplasms, including different subsets of B-cell acute lymphoblastic leukemia [38], [39] and the cervical carcinoma Hela cells. Similar to its role in conferring a survival advantage to CLL cells [10], [11], Ror1 was identified as a potent survival kinase in Hela cells [40]. Therefore, it is not surprising that we found that unstimulated B-lymphoid MM1 cells express low levels of Ror1 and that IL-6 upregulated Ror1 protein levels in these cells by activating Stat3. Constitutive Stat3 phosphorylation has been found in several solid tumors and hematologic malignancies [41], [42], [43], [44], and it is likely that ROR1 will be detected in these neoplasms as well.

Results obtained in unstimulated CLL cells were similar to those in IL-6-stimulated MM1 cells. EMSA confirmed that Stat3 binds to the ROR1 promoter, probe sets designed for the regions containing Stat3 putative binding sites determined that Stat3 binds to the ROR1 promoter at the same sites as in MM1 cells, and anti-Stat3 antibodies pulled down STAT3, ROR1, and Stat3-regulated gene DNA. Not all Stat3 putative binding sites were examined. Therefore, it is possible that Stat3 binding sites upstream of −429 might be operative. STAT3-shRNA downregulated STAT3 and ROR1 mRNA and Stat3 and Ror1 protein levels, suggesting that constitutively phosphorylated Stat3 activates ROR1 in CLL.

The interaction between Stat3 and Wnt signaling in CLL is not fully understood. Several investigators have reported that Stat3 induces Wnt5a expression [45], [46], [47]. Notably, Wnt5a binds to Ror1 [11]. Thus, it is possible that Stat3 activates the Wnt signaling pathway by inducing both ROR1 and Wnt5a expression in CLL cells. However, other interactions between Stat3 and the Wnt pathway exist in various cellular systems. In embryonic stem cells, the Wnt/β-catenin pathway upregulates Stat3 mRNA [48], and in thymocytes Wnt1 is overexpressed and induces serine phosphorylation of Stat3 without affecting tyrosine phosophorylation [49], suggesting an autocrine interaction between Stat3 and Wnt signaling. Whether a similar interaction exists in CLL remains to be determined.

## Materials and Methods

### B-cell CLL cell fractionation

PB cells were obtained from patients with CLL who were treated at The University of Texas M. D. Anderson Cancer Center Leukemia Clinic after Institutional Review Board approval and a written patient informed consent were obtained. The clinical characteristics of the patients whose PB samples were used in the study are presented in Table S1. To isolate low-density cells, PB cells were fractionated using Ficoll Hypaque 1077 (Sigma-Aldrich, St. Louis, MO). More than 90% of the PB lymphocytes obtained from these patients were CD19+/CD5+.

### Cell culture

Fractionated CLL cells were maintained in DMEM (Sigma-Aldrich) supplemented with 10% FBS (Hyclone, Logan, UT). The human multiple myeloma MM1 and the human embryonic kidney (HEK) 293FT cell lines were obtained from the American Type Culture Collection (Rockville, MD). MM1 cells were maintained in RPMI 1640 (Sigma-Aldrich), and 293FT cells were maintained in DMEM supplemented with 10% FBS, both in a humidified atmosphere of 5% CO_2_ at 37°C. Prior to analysis, MM1 cells were incubated with either recombinant human IL-6 (BioSource International, Camarillo, CA) or tissue culture medium.

### Western blot analysis

Western immunoblotting was performed as previously described [50]. Briefly, cell pellets were lysed in ice-cold Ripa buffer containing 1 mM sodium orthovanadate, 10 mM sodium fluoride, 100 µM EDTA, 5 mM β-glycerol phosphate, 1 µg/ml aprotinin, 0.4 mM benzamidine, 1 µg/ml antipain, 1 mM phenylmethylsulphonyl fluoride (PMSF), 1 µg/ml leupeptin, and 1 µg/ml soybean trypsin inhibitor. The lysed cell pellets were incubated on ice for 5 minutes and vortexed for 1 minute. Then, cell debris was eliminated by centrifugation at 14,000 rpm for 15 minutes, supernatants were harvested, and the protein concentration was determined using the Micro BCA protein assay reagent kit (Thermo Scientific, Pierce, Rockford, IL). Supernatant proteins were denatured by boiling for 5 minutes in sodium dodecyl sulfate (SDS) loading buffer, separated by SDS-polyacrylamide gel electrophoresis (SDS-PAGE), and transferred to a nitrocellulose membrane. Following transfer, equal loading was verified by Ponceau S staining. The membranes were blocked with 5% skim milk in Tris-buffered saline and incubated with the following antibodies: monoclonal mouse anti-human Stat3 (BD Biosciences, Palo Alto, CA; Cell Signaling Technology, Beverly, MA); monoclonal mouse anti-human phosphotyrosine Stat3, polyclonal rabbit anti-human phosphoserine Stat3, polyclonal rabbit anti-human Ror1 (Cell Signaling Technology), and monoclonal mouse anti-human β-actin (Sigma-Aldrich). After binding with horseradish peroxidase-conjugated secondary antibodies, blots were visualized with an ECL detection system (GE Healthcare, Amersham, Buckinghamshire, UK), and densitometry analysis was performed using the Epson Expression 1680 scanner (Epson America, Inc., Long Beach, CA). Densitometry results were normalized by dividing the numerical value of each sample signal by the numerical value of the signal from the corresponding loading control. In some experiments, the membranes were stripped by incubation with stripping buffer (62.5 mM Tris-HCl, pH = 6.7, 2% SDS, 100 mM β-mercaptoethanol) for 30 minutes at 50°C, washed, and re-probed with an antibody.

### Generation of luciferase reporter plasmids and site-specific mutagenesis

A 1.5-kb fragment of the human ROR1 promoter was generated by PCR using, as a template, genomic DNA isolated from PB low-density cells that were obtained from two patients with CLL (patients 13 and 14, Table S1). The DNA was purified by using the Wizard DNA Purification Kit (Promega, Madison, WI). Human ROR1 promoter PCR primers were designed in accordance with the sequence of the ROR1 5′-flanking region (Ensembl Resource, www.ensembl.org; Fig. 1). The ROR1 promoter PCR primers included a forward primer starting at bp −1173 (relative to the start of the first exon), a reverse primer starting at bp +349, and several smaller forward primers, as depicted in Table S2. The amplified fragments were attached to *BglII*- and *SacI*-digestion sites of the pGL4-basic vector (Promega) containing a luciferase reporter gene. The pGL4 vectors harboring ligation products were introduced into One Shot TOP10 chemically competent *E. coli* (Invitrogen, San Diego, CA). Fifty microliters of each transformed-bacteria batch was spread onto agar plates containing ampicillin (Invitrogen) and incubated overnight at 37°C. Single ampicillin-resistant colonies were microaspirated and analyzed following restriction enzyme digestion. The sequences of all constructs were verified by automated sequencing (Seqwright, Houston, TX).

The pGL4 basic reporter driven by 834-bp human Ror1 promoter was used for site-specific mutagenesis. Mutants at positions −133 and −418 (TT mutated to TG) were generated by using the QuickChange Site-Directed Mutagenesis kit (Stratagene) and confirmed by sequencing.

### Transfection of MM1 cells and luciferase assay

MM1 cells were transfected by electroporation with the Gene Pulser Xcell Electroporation System (Bio-Rad, Hercules, CA, USA). A total of 5×10^6^ cells, suspended in 0.2 ml serum-free OPTI-MEMI medium (Invitrogen, San Diego, CA, USA), were transfected with 2 µg of each of the specific reporter constructs. Luciferase activity was assessed 48 hours after transfection by using the Dual-Luciferase Reporter Assay System (Promega) and the SIRIUS luminometer V3.1 (Berthold Detection Systems, Pforzheim, Germany). The luciferase activity of each of the human ROR1 promoter constructs was determined by calculating the constructs' luciferase activity relative to the activity of the Renilla luciferase produced by the pRL-SV40 control vector and reporting the mean results of at least three separate experiments.

### RNA purification and qRT-PCR

RNA was isolated using an RNeasy purification procedure (Qiagen, Inc., Valencia, CA). RNA quality and concentration were analyzed with a NanoDrop spectrophotometer (ND-1000; NanoDrop Technologies, Wilmington, Delaware). Five hundred nanograms of total RNA was used in one-step qRT-PCR (Applied Biosystems, Foster City, CA), with the sequence detection system ABI Prism 7700 (Applied Biosystems) using TaqMan gene expression assay for ROR1 (Hs 00178178-M1), STAT3, Bcl2, Bcl-X_L_, c-Myc, Pim1, p21 (WAF1), Cyclin D1, and S18 according to the manufacturer's instructions. Samples were run in triplicate, and relative quantification was performed by using the comparative C_T_ method [14].

### Electrophoretic mobility shift assay (EMSA)

Non-denatured cellular nuclear extracts were prepared using the NE-PER extraction kit (Thermo Scientific, Pierce). Two micrograms of nuclear protein extracts were incubated with biotin-labeled ROR1 promoter DNA probes in binding buffer for 30 minutes on ice. All probes were synthesized by Sigma Genosys (The Woodlands, TX). The sequences of the probes used are as follows: probe 1, −143 CCG CGA CCG CTT TCT GCG AAG TCG GGG AGG −114; probe 2, −30 GGC TGG AGA GTT GGT GGA AAG TGA CAA GTT-1; probe 3, −429 TCT CTC AAC TCT TGG GTC CAA GAA GAA AGC −400; and probe 4, −1053 TGA CTC TTT TTA GTG TCA ATG GAG GAT TTG −1024. Following incubation, the samples were separated on a 5% polyacrylamide gel in Tris-borate EDTA, transferred onto a nylon membrane, and fixed on the membrane by ultraviolet cross-linking. The biotin-labeled probe was detected with strepavidin-horseradish peroxidase (Gel–Shift Kit; Panomics, Fremont, CA). The competition control consisted of up to 7-fold excess unlabeled cold probe combined with biotin-labeled probes. To determine the effect of antibodies on protein-DNA binding, 1 µg monoclonal mouse anti-human Stat3 (BD Bioscience; Cell Signaling Technology) or mouse anti-human phosphoserine Stat3 (Cell Signaling Technology) antibody was incubated with the nuclear extracts for 30 minutes on ice prior to the addition of the biotin-labeled DNA probe. The isotypic controls consisted of mouse immunoglobulin (Ig) G1 (BD Bioscience).

### ChIP assay

The ChIP assay was performed using a SimpleChIP Enzymatic Chromatin IP Kit (Cell Signaling Technology) in accordance with the manufacturer's instructions. Briefly, cells were cross-linked with 1% formaldehyde for 10 minutes at room temperature and then harvested and incubated on ice for 10 minutes in lysis buffer. Nuclei were pelleted and digested by micrococcal nuclease. Following sonication and centrifugation, sheared chromatin was incubated with anti-STAT3 or rabbit serum (negative control) overnight at 4°C. Then, protein G beads were added and the chromatin was incubated for 2 hours in rotation. An aliquot of chromatin that was not incubated with an antibody was used as the input control sample. Antibody-bound protein/DNA complexes were eluted and subjected to PCR analysis. The primers to amplify the human STAT3 promoter were F: 5′-CCG AAC GAG CTG GCC TTT CAT-3 and R: 5′-GGA TTG GCT GAA GGG GCT GTA-3, which generated an 86-bp product; primers to amplify the WAF/p21 promoter were F: 5′-TTG TGC CAC TGC TGA CTT TGT C-3 and R: 5′-CCT CAC ATC CTC CTT CTT CAG GCT-3, which generated a 303-bp product; and primers to amplify the c-Myc promoter were F: 5′-TGA GTA TAA AAG CCG GTT TTC-3 and R: 5′-AGT AAT TCC AGC GAG AGG CAG-3, which generated a 63-bp product. The primer sets used to amplify ROR1 promoter putative Stat3 binding sites were as follows: set 1, −195 TTT GAG GAG TGT GGG GGA GGG and GTT GAG AGG CTG CAG CAG AGG−85; set 2, −105 GTT GAG AGG CTG CAG CAG AGG and GGC TGG AGA GTT GGT GGA AAG−10; set 3, −509 GGC AGT ACA GAT GAC ATT GAA and TTG GGT CCA AGA AGA AAG CAA−400; set 4, −1173 TGG CTA CCC CAT GTC TGT GAG and GGA GGA TTT GTG AGC AAA TGC−1013; and set 5, 1595 CAT TCG GAA GAC AAA AAC ACC and GGC AGT ACA GAT GAC ATT GAA−489. The human RPL30 gene primers were provided by Cell Signaling Technology. PCR products were resolved on 1.8% agarose gels.

### Stat3 and ROR1 siRNA transfection

MM1 and CLL cells were transfected by electroporation as described above. Knockdown of endogenous Stat3 was performed using the pre-designed siRNA and scrambled siRNA from Ambion Applied Biosystems. The siRNA sequences used to target exon 14 and 15 of the human STAT3 gene were antisense 5′-GGG AAG CAU CAC AAU UGG Ctc-3′ and sense 5′-GCC AAU UGU GAU GCU UCC Ctt-3′. SiRNA at a final concentration of 50 nM was also co-transfected with luciferase reporter plasmids into MM1 cells. Transfectants were maintained in the complete medium for 48 hours before being used in the luciferase activity assay and in qRT-PCR analysis. The siRNA sequences used to target the human ROR1 gene were antisense 5′-AGA CUU AAA GUU UUC AUC CAA-3′ and sense 5′-GGA UGA AAA CUU UAA GUC UTT. 30nM of siRNA was mixed with siPORT *Neo*FX Transfection Agent (Ambion) and transfected by electroporation into CLL cells. Transfected cells were maintained in co-culture with mesenchymal stroma cells and qRT-PCR of ROR1 was performed at 72 hours after transfection.

### Preparation of lentivirus STAT3-shRNA and infection of cells

HEK 293FT cells were co-transfected with GFP-lentivirus STAT3 shRNA or GFP-lentivirus empty vector and the packaging vectors pCMVδR8.2 and pMDG (generously provided by Dr. G. Inghirami, Torino, Italy) using the superfect transfection reagent (Qiagen, Inc.). 293FT cell culture medium was changed after 16 hours and collected after 48 hours. The culture medium was filtered through a 45-µm syringe filter to remove floating cells, the lentivirus was concentrated by filtration through an Amicon Ultra centrifugal filter device (Milipore, Billerica, MA), and the concentrated supernatant was used to infect CLL cells. CLL cells (5×10^6^/ml) were incubated in 6-well plates (Becton Dickinson, Franklin Lakes, NJ) in 2 ml DMEM supplemented with 10% FBS and transfected with 100 µl of viral supernatant. Polybrene (10 ng/ml) was added to the viral supernatant at a ratio of 1∶1000 (v/v). Transfection efficiency was measured after 48 hours and was found to range between 45% and 60% (calculated on the basis of the ratio of propidium iodide (PI)-negative/GFP-positive cells). RNA was isolated and prepared for qRT-PCR analysis as previously described [14].

## Supporting Information

Table S1 Patient characteristics.
(0.05 MB DOC)

Table S2 The 5′ and 3′ primers used for generating the luciferase reporter constructs of the human ROR1 promoter.
(0.04 MB DOC)
