# Supplementary material for: Stat3 Activates the Receptor Tyrosine Kinase Like Orphan Receptor-1 Gene in Chronic Lymphocytic Leukemia Cells
Source: PLoS One. 2010 Jul 29;5(7):e11859. doi: 10.1371/journal.pone.0011859 (PMC2912280; doi:10.1371/journal.pone.0011859)
Supplement: Table S1 — Patient characteristics. (0.05 MB DOC) [file pone.0011859.s001.doc]

**Table S1:** Patient characteristics.

| **CLL  pt. #** | **Sex** | **Age (yr)** | **WBC (10^9^/I)** | **Lymph. %** | **Hb. (g/dl)** | **Plts. (10^9^/I)** | **Rai Stage** | **CD38+/ 19+ %** | **β_2_M (mg/dl)** | **V_H_ mutation** | **ZAP 70 %** | **Status** | **Cytogenetics** | **Previous treatment** |
| --- | --- | --- | --- | --- | --- | --- | --- | --- | --- | --- | --- | --- | --- | --- |
| 1 | M | 82 | 102.4 | 85 | 13.3 | 154 | 2 | 4.1 | 4.3 | N | 5.7 | Alive | t;12 | Rituxan+GM |
| 2 | F | 85 | 243.7 | 96 | 11.3 | 136 | 0 | not done | 3.1 | Y | 6 | Alive | del 13q |  |
| 3 | M | 34 | 39 | 81 | 13.9 | 147 | 0 | 12.5 | 2.1 | Y | 5.2 | Alive | not done | Fludarabine; CTX+Steroids |
| 4 | F | 66 | 138.5 | 83 | 11.4 | 342 | 1 | 87.8 | 4.9 | N | 24.8 | Alive | del 13q | CTX+ Rituxan |
| 5 | M | 61 | 292.8 | 96 | 9.1 | 41 | 4 | 2 | 3.6 | N | ND | Dead | del 13q | FCR |
| 6 | F | 40 | 3.13 | 90 | 12 | 170 | 2 | not done | 4.3 | Y | 0 | Alive | del 13q |  |
| 7 | F | 73 | 160 | 90 | 12.1 | 146 | 1 | 1.1 | 4 | N | 44.6 | Alive | del 13q | Chlorambucil |
| 8 | M | 58 | 84.1 | 62 | 13.4 | 111 | 1 | not done | 2.5 | Y | 10.2 | Alive | del 13q |  |
| 9 | F | 41 | 213.3 | 98 | 10.8 | 129 | 3 | 0.2 |  | Y | 2.3 | Alive | del 13q |  |
| 10 | M | 47 | 22.8 | 87 | 14.3 | 132 | 1 | not done | 2 | Y | 13.6 | Alive | del 13q |  |
| 11 | F | 73 | 177.9 | 94 | 11.4 | 192 | 2 | 30.8 | 3.1 | N/A | 54.2 | Alive | t12 |  |
| 12 | F | 67 | 84.1 | 92 | 10 | 138 | 3 | 30.5 | 10 | Y | 4 | Alive | del 13q |  |
| 13 | F | 55 | 77.5 | 96 | 13.4 | 171 | 0 | not done | 2.2 | Y | 40.7 | Alive | del 13q |  |
| 14 | F | 70 | 111.6 | 94 | 13.5 | 127 | 1 | 31.6 | 5.1 | Y | 1.25 | Alive | 17p |  |

**Abbreviations:** Yr., years; WBC, white blood cells; lymph., lymphocytes; Hb., hemoglobin; Plts., platelets; β_2_M, β_2_ microglobulin; M, male; F, female; N/A, not available; V_H_ mutation, hypermutation of the immunoglobulin heavy chain gene presented as N (negative; if % derivation from the germline sequence is < 2%) or Y (positive, if % derivation from germline sequence is > 2%); GM, granulocyte-macrophage colony-stimulating factor; CTX, cyclophosphamide; FCR, cyclophosphamide, fludarabine, and Rituxan.
