# Supplementary material for: Stat3 Activates the Receptor Tyrosine Kinase Like Orphan Receptor-1 Gene in Chronic Lymphocytic Leukemia Cells
Source: PLoS One. 2010 Jul 29;5(7):e11859. doi: 10.1371/journal.pone.0011859 (PMC2912280; doi:10.1371/journal.pone.0011859)
Supplement: Table S2 — The 5′ and 3′ primers used for generating the luciferase reporter constructs of the human ROR1 promoter. (0.04 MB DOC) [file pone.0011859.s002.doc]

**Table S2:** The 5’ and 3’ primers used for generating the luciferase reporter constructs of the human ROR1 promoter.

| ROR1 +177: | GCG | GAG | CTC | TGC | AGC | CAG | AGG | GCT | GGG | AAG |
| --- | --- | --- | --- | --- | --- | --- | --- | --- | --- | --- |
| ROR1 +-32: | GCG | GAG | CTC | TGG | GCT | GGA | GAG | TTG | GTG | GAA |
| ROR1 -195: | GCG | GAG | CTC | TTT | GAG | GAG | TGT | GGG | GGA | GGG |
| ROR1 -360: | GCG | GAG | CTC | CCA | GAG | TCA | CAC | AGC | TAA | CAG |
| ROR1 -666: | GCG | GAG | CTC | TCG | AAA | GAT | TCA | GAA | GGC | CTG |
| ROR1 -834: | GCG | GAG | CTC | AGG | CAG | TCT | CTA | CAG | GGA | AAG |
| ROR1 -1173: | GCG | GAG | CTC | TGG | CTA | CCC | CAT | GTC | TGT | GAG |
| ROR1 +349-3: | GCG | AGA | TCT | CAG | AAC | ATC | CAC | GGG | CTC | TTC |
| ROR1 -340-3: | GCG | AGA | TCT | CTG | TTA | GCT | GTG | TGA | CTC | TGG |
| ROR1 -10-3: | GCG | AGA | TCT | CTT | TCC | ACC | AAC | TCT | CCA | GCC |

Underlined are the restriction enzyme sites.
